# Syndemic Psychosocial Conditions among Youth Living with HIV: a Latent Class Analysis

**DOI:** 10.1007/s10461-024-04427-7

**Published:** 2024-07-17

**Authors:** John Mark Wiginton, K. Rivet Amico, Lisa Hightow-Weidman, Patrick Sullivan, Keith J. Horvath

**Affiliations:** 1 Division of Infectious Diseases and Global Public Health, Department of Medicine, University of California-San Diego, 9500 Gilman Drive, La Jolla, CA 92093-0507 USA; 2 Department of Health Behavior and Health Education, School of Public Health, University of Michigan, Ann Arbor, USA; 3 Institute on Digital Health and Innovation, College of Nursing, Florida State University, Tallahassee, USA; 4 Department of Epidemiology, Rollins School of Public Health, Emory University, Atlanta, USA; 5 Department of Psychology, San Diego State University, San Diego, USA

**Keywords:** Syndemic, Youth living with HIV, HIV care continuum, Psychosocial conditions

## Abstract

Drug use, mental distress, and other psychosocial factors threaten HIV care for youth living with HIV (YLWH). We aimed to identify syndemic psychosocial patterns among YLWH and examine how such patterns shape HIV outcomes. Using baseline data from 208 YLWH enrolled in an HIV treatment adherence intervention, we performed latent class analysis on dichotomized responses to 9 psychosocial indicators (enacted HIV stigma; clinical depression and anxiety; alcohol, marijuana, and illicit drug misuse; food and housing insecurity; legal history). We used multinomial logistic regression to assess latent class-demographic associations and the automatic Bolck-Croon-Hagenaars method to assess HIV outcomes by class. Mean age of participants was 21 years; two thirds identified as cis male, 60% were non-Hispanic Black, and half identified as gay. Three classes emerged: “Polydrug-Socioeconomic Syndemic” (*n* = 29; 13.9%), “Distress-Socioeconomic Syndemic” (*n* = 35, 17.1%), and “Syndemic-free” (*n* = 142, 69.0%). Older, unemployed non-students were overrepresented in the “Polydrug-Socioeconomic Syndemic” class. Missed/no HIV care appointments was significantly higher in the “Polydrug-Socioeconomic Syndemic” class (81.4%) relative to the “Syndemic-free” (32.8%) and “Distress-Socioeconomic Syndemic” (31.0%) classes. HIV treatment nonadherence was significantly higher in the “Polydrug-Socioeconomic Syndemic” class (88.5%) relative to the “Syndemic-free” class (59.4%) but not the “Distress-Socioeconomic Syndemic” class (70.8%). Lack of HIV viral load suppression was non-significantly higher in the “Polydrug-Socioeconomic Syndemic” class (29.7%) relative to the “Syndemic-free” (16.2%) and “Distress-Socioeconomic Syndemic” (15.4%) classes. Polydrug-using, socioeconomically vulnerable YLWH are at risk for adverse HIV outcomes, warranting tailored programming integrated into extant systems of HIV care.

## Introduction

HIV continues to impact youth in the United States (US). According to the Centers for Disease Control and Prevention (CDC), nearly 20% of new HIV diagnoses in 2021 occurred among individuals aged 13 to 24 years, and recent research indicates that most of these diagnoses occurred among youth of color [1, 2]. CDC data also demonstrate that youth living with HIV (YLWH) encounter difficulties across the HIV care continuum: 20% receive no HIV care, 45% are unretained in care, and 35% are virally unsuppressed [3]. Moreover, a recent systematic review and meta-analysis found that 47% of YLWH in North America were nonadherent to HIV treatment [4]. Supporting YLWH, especially racial/ethnic minority YLWH, is critical to their engagement in the HIV care continuum, their living well with HIV, and reducing HIV transmission to end the HIV epidemic in the US [5].

Psychosocial conditions may underlie observed HIV care continuum disparities experienced by YLWH [1, 6]. Specifically, substance misuse (e.g., alcohol, marijuana, illicit drugs) [7–11], mental health concerns (e.g., depression, anxiety) [7, 8, 12], enacted HIV stigma (e.g., from social network, healthcare providers) [13–15], poverty (e.g., food and housing insecurity) [10, 16–19], and criminal justice involvement (e.g., transitioning to or [re]establishing care following release from incarceration) [20, 21] have all been linked to poor retention in HIV care and nonadherence to HIV treatment (or elevated HIV viral load, indicative of nonadherence to HIV treatment). Youth who experience these conditions may be especially vulnerable to their impact because youth have yet to mature socially, emotionally, cognitively, or physically [22], and because youth may lack access to resources to mitigate the effects of these conditions [23].

Moreover, psychosocial conditions often co-occur, further exacerbating health outcomes. To describe such a scenario, Singer [24, 25] proposed the term *syndemic*, denoting “a set of closely interrelated, endemic and epidemic conditions,” an “interrelated complex of health and social crises,” and the “synergistic interaction of diseases and social conditions” that contribute to disproportionate disease burden [25, 26]. Additive and latent variable (e.g., latent class analysis [LCA]) approaches are often used to study syndemic health states. Additive approaches typically involve regressing an outcome on a syndemic index modeled continuously or categorically. For example, among YLWH, Kuhns and colleagues [27] showed a decreasing likelihood of HIV treatment adherence and HIV viral load suppression with an increasing number of co-occurring conditions. In various research with adults living with HIV, the likelihood of HIV care appointment attendance, HIV treatment adherence, and HIV viral load undetectability decreased as the number of co-occurring conditions increased [28–35].

LCA approaches involve identifying patterns of co-occurring conditions that reflect underlying subgroups, or classes, of people with similar profiles in a heterogeneous population [36, 37]. These approaches provide complementary but more nuanced findings than additive approaches. For example, Hotton et al. [38] identified two syndemic patterns (“higher adversity,” “lower adversity”) among trans women of color living with HIV. Participants in the “higher adversity” class, which featured legal history, housing instability, and other socioeconomic insecurities, were less likely to have recently utilized HIV care and less likely to be virally suppressed. Traynor et al. [39] identified three syndemic patterns (“lower barriers,” “higher barriers/history of abuse and intimate partner violence,” “higher barriers/history of discrimination, abuse, and intimate partner violence”) among substance-using people living with HIV (PLWH). Those in the “higher barriers” class that featured discrimination, abuse, and violence did not benefit from a patient navigation intervention to increase HIV care engagement and viral suppression, unlike participants in the other classes. Robinson et al. [40] identified four syndemic patterns (“moderate substance use/mental illness,” “high mental illness,” “moderate substance and mental illness/high familial conflict non-negotiation,” “high substance use/high mental illness”) among African-Americans living with HIV in Baltimore, Maryland. Participants in the “high substance use/high mental illness” class were the least likely to have achieved HIV viral suppression.

Whether defined by number or pattern, psychosocial syndemics exist among PLWH and shape HIV outcomes. However, there has been limited research examining syndemics among YLWH [e.g., 27]. We sought to fill this gap with the present study. Specifically, we aimed to identify classes of co-occurring psychosocial conditions among YLWH, determine demographic correlates of class membership, and assess the extent to which HIV care continuum outcomes varied across classes. Findings can inform future research on syndemics among YLWH, as well as inform intervention and programming efforts to identify YLWH with the greatest need for HIV care support and comprehensive wraparound services.

## Methods

### Data Source, Participants, and Procedures

Data were drawn from a baseline assessment of YLWH enrolled in a randomized controlled trial of a web-based intervention called “YouTHrive,” which aimed to improve ART adherence and HIV treatment outcomes among YLWH in six urban areas in the US (Atlanta, Chicago, Houston, New York, Philadelphia, Tampa). Detailed methods have appeared elsewhere [41]. Briefly, enrollment began in August 2019 and ended in May 2022. Eligibility criteria included (a) being aged 15–24 years at enrollment, (b) living with HIV, (c) residing in one of the aforesaid six urban areas with availability to meet in-person for baseline and follow-up visits, (d) being currently prescribed antiretroviral therapy (ART; documented with prescription or pill bottle), (e) being English-speaking, (f) expecting to have continuous internet access and SMS messaging for the intervention period, (g) having an email address to use during the study period (or being willing to create one), (h) not being a member of an iTech Youth Advisory Board, and (i) not being enrolled in another ART adherence intervention research study. Pre-COVID, an additional eligibility criterion was required: that participants have either (a) a past-year detectable viral load test result while on ART for 3 or more months, (b) 1 or more missed HIV care appointments in the past 12 months, (c) no HIV care visit in the previous 6 months, or (d) < 90% ART adherence in the previous 4 weeks. This criterion was assessed through medical chart verification or self-report and applied to the one third of participants enrolled pre-COVID.

Eligible participants were recruited from HIV clinics or through community outreach efforts (e.g., targeted social media advertisements) and provided information on HIV treatment-related outcomes, mental health, HIV stigma, demographic characteristics, and other domains via a computer-assisted survey instrument. Participants received $50 for survey completion. This study is registered as a clinical trial (Clinical Trials # NCT03149757) and was approved by the University of North Carolina-Chapel Hill Institutional Review Board (as the single institutional review board); parental consent was waived for participants aged 15–17 years.

### Measures

#### Latent Class Indicators of Syndemic Factors

We used items developed by Earnshaw and colleagues to assess experiences of enacted HIV stigma [42, 43]. In response to the same prompt (“How often have people treated you this way in the past because of your HIV status?”), participants responded to 9 items (e.g., “Community/social workers have denied me services.”) on a 5-point Likert scale, ranging from 1 = Never to 5 = Very often. We calculated average scores and dichotomized them at 2 (set a priori) to separate those whose average score indicated often experiencing enacted stigma (> 2; i.e., somewhat often, often, very often; coded 1) from those whose average score did not indicate this (≤ 2, i.e., never or not often; coded 0).

Clinical depression and anxiety were assessed with the Patient Health Questionnaire-8 (PHQ-8) [44] and the Generalized Anxiety Disorder Assessment-7 (GAD-7) [45], respectively. Using one stem (“Over the past 2 weeks, how often have you been bothered by any of the following problems?”), PHQ-8 and GAD-7 items assessed the frequency of having experienced depressive (e.g., “Little interest in pleasure or doing things”) and anxiety symptoms (e.g., “Feeling nervous, anxious, or on edge”). Participants responded on a 4-point Likert scale for each, ranging from 0 = Not at all to 3 = Nearly every day. Items were summed on each scale to yield a composite score, with scores ≥ 10 indicative of a clinical level of the assessed mental health concern.

Alcohol misuse, marijuana misuse, and illicit drug misuse (cocaine, methamphetamines, inhalants, downers, hallucinogens, opioids) were assessed with the Alcohol, Smoking and Substance Involvement Screening Test [46]. For those endorsing ever having used a given substance, 6 items (e.g., “During the past three months, how often have you failed to do what was normally expected of you because of your use of [substance]?”) assessed use over the previous 3 months. Likert-scale response options for the first 4 items ranged from 0 = Never to 6 = Daily or almost daily (item-dependent; for some, the Daily or almost daily option was scored as 7 or 8). Response options to the final 2 items included 0 = No, never; 3 = Yes, but not in the past 3 months; and 6 = Yes, in the past 3 months. Items were summed to yield a compose score. Total scores of ≥ 11 on the alcohol items and ≥ 4 on all other items were indicative of misuse. We dichotomized based on these thresholds. As misuse of individual illicit drugs was low, we created a global illicit drug misuse variable: a score of 4 or higher on any of the aforementioned illicit drugs was considered illicit drug misuse.

Food insecurity was assessed with one item asking about the frequency participants or their family had to reduce meal sizes or skip meals due to inadequate food or money. Response options ranged from 1 = Almost every week to 4 = Did not have to skip or cut the size of meals. We dichotomized this item, coding any endorsement (1–3) as 1 and no endorsement (4) as 0. Unstable housing was assessed with several dichotomous items assessing whether participants had spent at least one night in a shelter, in a public place not intended for sleeping, on the street or anywhere outside, temporarily doubled up with a friend or family member, in a temporary housing program, or in a welfare or voucher hotel/motel. We created a binary variable based on these responses, with endorsement of any being coded 1 and non-endorsement of all coded 0. Legal involvement was assessed with two dichotomous items asking whether participants had ever been (a) arrested or (b) put in jail, prison, or juvenile detention. Endorsement of either item was coded 1; non-endorsement of both was coded 0.

#### Outcomes

We assessed HIV care engagement with two items: “In the last 12 months, how many scheduled appointments did you have?” and “In the last 12 months, how many of your scheduled appointments did you miss because you didn’t show or forgot?” Participants could write-in a numerical response for each. We created a continuous outcome for missed HIV care appointments (based on responses to the second item), and a binary outcome for any missed HIV care appointments or having none scheduled at all (i.e., those who responded 0 to the first item and > 0 to the second item). We assessed HIV treatment adherence with one item: “In the last 30 days, on how many days did you miss at least one dose of any of your HIV medicines?” Participants could write-in a numerical response. We used this raw response as a continuous outcome, and we used dichotomized responses (i.e., any response > 0 coded 1) as a binary outcome. We assessed viral load via biospecimen testing carried out at participating HIV clinics and created a dichotomous unsuppressed viral load variable (> 200 copies/mL).

#### Other Variables

Demographic variables included age in years (continuous and age group [21–24 years vs. 15–20 years]), gender identity (cis man, cis woman, gender-diverse), race/ethnicity (non-Hispanic Black, non-Hispanic other race, Hispanic), sexual identity (gay, bisexual or other non-gay identity, straight), education (enrolled in school or not), employment status (unemployed and non-student vs. employed and/or student), relationship status (partnered vs. single), and insurance status (uninsured vs. insured).

### Statistical Analysis

We computed descriptive statistics for variables of interest and assessed missingness. We performed LCA on responses to the 9 syndemic factors. Models with 2–4 classes were considered, and different sets of starting values were used to assess model identification. We selected the best-fitting model using Akaike’s Information Criterion (AIC), sample size-adjusted Bayesian Information Criterion (SSA-BIC), integrated classification likelihood-BIC (ICL-BIC), adjusted Vuong-Lo-Mendell-Rubin likelihood ratio test (aVLMR-LRT), bootstrapped likelihood ratio test (BLRT), log-likelihood value, entropy statistic of class delineation, lowest average classification probability, lowest class prevalence, parsimony, and scientific interpretation [37, 47–55]. We used a full maximum likelihood estimator under the assumption data were missing at random to derive estimates based on all available data while also accounting for missingness [56, 57]. We examined standardized bivariate residuals of expected versus observed responses to indicator pairs to assess conditional independence, with plans to allow dependent items (z>|1.96|), if present, to correlate [58]. We considered indicator probabilities of < 0.30 as low, ≥ 0.30 < 0.70 as moderate, and ≥ 0.70 as high.

Covariate associations with class membership were examined via multinomial logistic regression using the automatic 3-step method to correct for potential classification error in latent class assignment [59, 60]. Coefficients were exponentiated to generate unadjusted odds ratios (OR); Wald tests, with statistical significance set at 0.05 and 95% confidence intervals (CI), were also calculated and examined. We used the automatic Bolck-Croon-Hagenaars (BCH) method – which independently estimates outcome prevalence or class mean while simultaneously estimating the measurement model [59] – to examine HIV outcomes across classes. We assessed class differences in outcomes with chi-square tests. Descriptive analyses and data preparation were conducted in Stata Version 15 (StatCorp, College Station, TX, USA). LCA and related procedures were conducted in Mplus Version 8.5 (Muthen & Muthen, Los Angeles, CA).

## Results

### Sample Characteristics

Out of 208 participants enrolled at baseline, 206 provided complete LCA indicator data and were included in the analysis. Mean age was 21 years, two thirds (*n* = 139) identified as a cisgender man, and 59.7% (*n* = 123) were non-Hispanic Black. Roughly half (*n* = 102) identified as gay, and 3 in 10 (*n* = 61) were partnered. Over half (*n* = 110) were in school, and of these, the majority (*n* = 78) reported currently receiving post-high school education. Almost 1 in 5 (*n* = 37) were unemployed non-students and were also uninsured (Table 1).

**Table 1:** YLWH enrolled in an ART-adherence intervention in six US urban areas, 2019–2022 (*N* = 206)

	Total (*N* = 206)
Age (in years, continuous)	
Mean (SD), median (IQR)	21.2 (2.3), 22 (20–23)
Age group, *n* (%)	
15–20	74 (35.9)
21–24	132 (64.1)
Gender identity, *n* (%)	
Cisgender man	139 (67.5)
Cisgender woman	49 (23.8)
Gender-diverse	17 (8.3)
*Transfeminine or transwoman*	*4 (1.9)*
* Genderqueer*	*7 (3.4)*
* Other, multiply-identified*^a^	*6 (2.9)*
Missing/unknown	1 (0.5)
Race/ethnicity, *n* (%)	
Non-Hispanic Black	123 (59.7)
Non-Hispanic Other	28 (13.6)
*White*	*9 (4.4)*
* American Indian, Alaska Native*	*1 (0.5)*
* Asian, Asian-American*	*3 (1.5)*
* Multiracial*	*11 (5.3)*
* Other, unspecified race*	*4 (1.9)*
Hispanic	48 (23.3)
*Black*	*7 (3.4)*
* White*	*24 (11.7)*
* American Indian, Alaska Native*	*6 (2.9)*
* Native Hawaiian, Other Pacific Islander*	*1 (0.5)*
* Multiracial*	*4 (1.9)*
* Other, unspecified race*	*6 (2.9)*
Ethnicity unknown/missing	
Black	2 (1.0)
Asian, Asian-American	1 (0.5)
Multiracial	1 (0.5)
Race/ethnicity missing/unknown	3 (1.5)
Sexual identity, *n* (%)	
Gay	102 (49.5)
Bisexual, other non-gay minority identities^b^	47 (22.8)
Straight	54 (26.2)
Missing/unknown	3 (1.5)
Education, n (%)	
Enrolled in school	110 (53.4)
Not enrolled in school	94 (45.6)
Missing/unknown	2 (1.0)
Employment status, *n* (%)	
Employed and/or in school	167 (81.1)
Unemployed and not in school	37 (18.0)
Missing/unknown	2 (1.0)
Relationship status, *n* (%)	
Single	141 (68.4)
Partnered	61 (29.6)
Missing/unknown	4 (1.9)
Insurance status, *n* (%)	
Insured	151 (73.3)
Uninsured	37 (18.0)
Missing/unknown	18 (8.7)

YLWH: youth living with human immunodeficiency virus; ART: antiretroviral treatment; US: United States; SD: standard deviation; IQR: interquartile range; ^a^With 2 participants who identified as male & genderqueer, 2 who identified as genderqueer & transwoman, 1 who was assigned the female sex at birth but currently identifies as male but not trans male, and 1 who was assigned the male sex at birth but currently identifies as female but not trans female; ^b^With 6 participants who identified as gay & bisexual, 3 who identified as bisexual & straight, 3 who identified as pansexual, 1 who identified as gynosexual, and 1 who identified as non-conforming

Of the 9 syndemic factors examined, marijuana misuse was the most endorsed (48.1%; *n* = 99), followed by food insecurity (27.7%; *n* = 57), legal involvement (26.2%; *n* = 54), clinical depression and anxiety (both 23.8%; *n* = 49), unstable housing and alcohol misuse (both 18.4%; *n* = 38), and illicit drug misuse and enacted stigma (both 11.7%; *n* = 24). More than three quarters (*n* = 157) of participants reported the presence of ≥ 1 syndemic factor, with 24.3% (*n* = 50) reporting 1 factor only, 19.4% (*n* = 40) reporting 2 factors, and 32.5% (*n* = 67) reporting ≥ 3.

More than a third (*n* = 72) of participants either missed or did not schedule any HIV care appointments in the past year. The average number of missed HIV care appointments for those who had scheduled any was 0.77 (SD = 1.4; range = 0–9); 5 participants reported they had not scheduled any appointments at all during the past year. Almost two thirds (*n* = 130) reported at least 1 day in the prior 30 in which they had missed at least 1 dose of HIV treatment. Average number of days of having missed ≥ 1 doses was 2.7 (SD = 4.6; range = 0–30). Biospecimen testing showed 16.0% (*n* = 33) had unsuppressed HIV viral load.

### Latent Class Enumeration

The best log-likelihood was successfully replicated several times for each unconditional 2–4 class solution using numerous sets of increasingly high starting values, suggesting model identifiability for each solution. The AIC, SSA-BIC, and ICL-BIC decreased from the 2- to the 3-class model but increased from the 3- to the 4-class model, with an elbow depicted at 3 classes. The BLRT indicated that the 2-class model fit the data significantly better than the 1-class model (BLRT = 161.49, df = 10, *p* < 0.001) and that the 3-class model fit the data significantly better than the 2-class model (BLRT = 43.27, df = 10, *p* < 0.001), but that the 4-class model did not fit the data significantly better than the 3-class model (BLRT = 19.93, df = 10, *p* = 0.364). Similarly, the aVLMR-LRT indicated that the 2-class model fit the data significantly better than the 1-class model (aVLMR-LRT = 158.52, df = 10, *p* < 0.001) and that the 3-class model fit the data marginally better than the 2-class model (aVLMR-LRT = 42.47, df = 10, *p* = 0.062), but that the 4-class model did not fit the data significantly (or marginally) better than the 3-class model (aVLMR-LRT = 19.57, df = 10, *p* = 0.484). Entropy and average latent class probabilities exceeded 0.80 for the 3-class model, indicating good class separation, and lowest class prevalence (14.3%) exceeded the optimal threshold of 5 ~ 9%. Weighing this information, we selected the 3-class model (Table 2).

**Table 2:** Fit indices and model characteristics of unconditional LC solutions of syndemic factors among YLWH in the US, 2019–2022 (*N* = 206)

	2 classes	3 classes	4 classes
Log Likelihood (improvement)	-867.38	**-845.75**	-835.78
Number of free parameters	19	**29**	39
AIC	1772.76	**1749.49**	1749.56
SSA-BIC	1775.79	**1754.12**	1755.78
ICL-BIC	1780.28	**1760.30**	1763.56
aVLMR-LRT statistic, df, *p*-value	158.52, 10, < 0.001	**42.47, 10, 0.062**	19.57, 10, 0.484
BLRT statistic, df, *p*-value	161.49, 10, < 0.001	**43.27, 10, < 0.001**	19.93, 10, 0.364
Entropy	0.78	**0.85**	0.88
Lowest average latent class probability	0.94	**0.86**	0.86
Lowest class prevalence	31.9%	**14.3% (13.9%)** ^**1**^	3.8%

LC: latent class; YLWH: youth living with human immunodeficiency virus; US: United States; AIC: Akaike’s Information Criterion; SSA-BIC: sample size-adjusted Bayes’ Information Criterion; ICL-BIC: Integrated Classification Likelihood-BIC; aVLMR-LRT: adjusted Vuong-Lo-Mendell-Rubin likelihood ratio test; BLRT: bootstrapped likelihood ratio test; df: degrees of freedom

Bolded column selected as the optimal class solution

^1^After modeling residual covariance, lowest class prevalence dropped to 13.9%

**Fig. 1:**
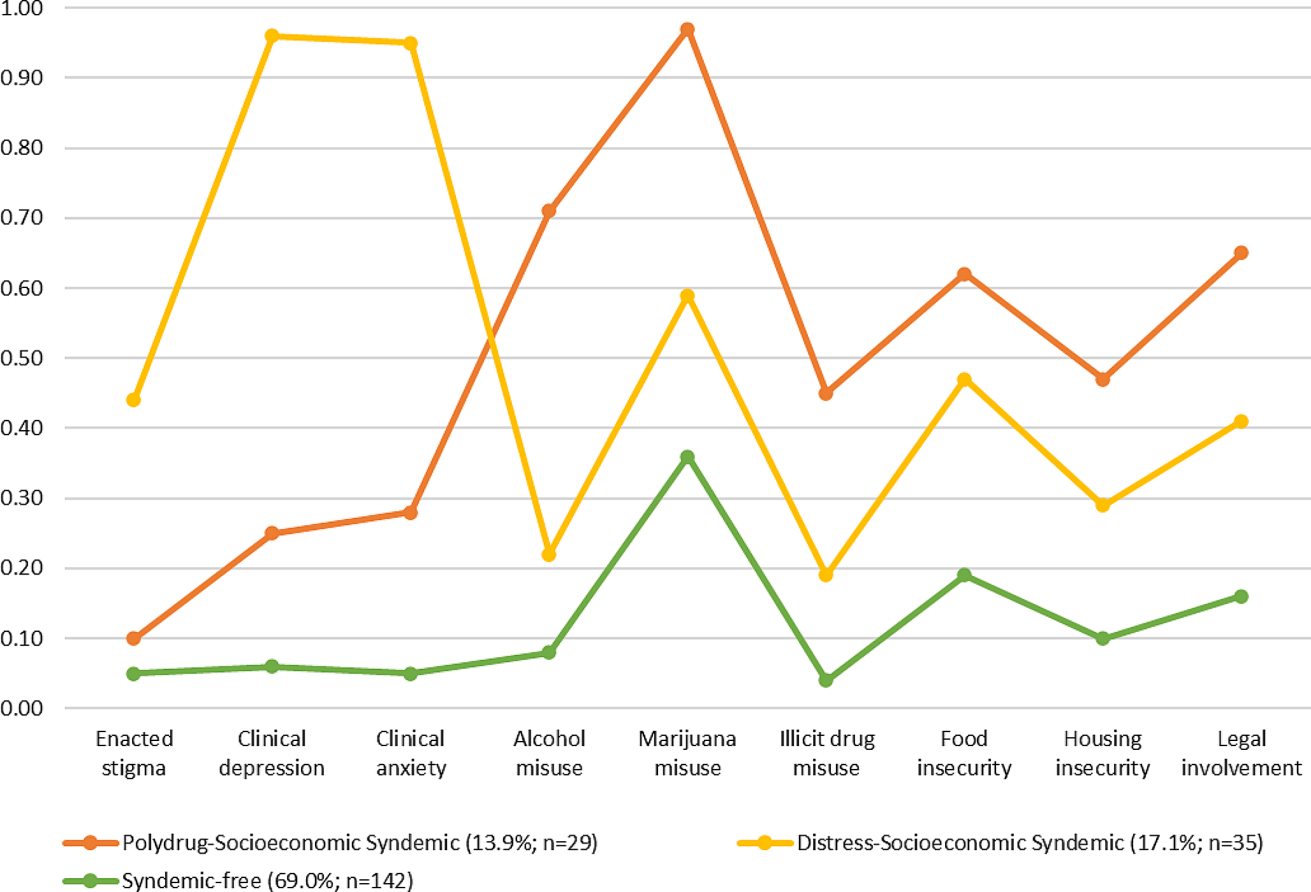
The 3-model solution of syndemic psychosocial conditions among youth living with HIV in the United States, 2019–2022 (*N* = 206)

Class 1, “Polydrug-Socioeconomic Syndemic” (13.9%, *n* = 29), featured high probabilities of alcohol and marijuana misuse (0.71–0.97), moderate probabilities of illicit drug misuse, food and housing insecurity, and legal involvement (0.45–0.65), and low probabilities of other indicators (0.10–0.28). Class 2, “Distress-Socioeconomic Syndemic” (17.1%, *n* = 35), featured high probabilities of clinical depression and anxiety (0.95–0.96), moderate probabilities of enacted HIV stigma, marijuana misuse, food insecurity, and legal involvement (0.41–0.59), and low probabilities of other indicators (0.19–0.29). Class 3, “Syndemic-free” (69.0%, *n* = 142), featured a moderate probability of marijuana misuse (0.36) and low probabilities of other indicators (0.04–0.19; Fig. 1).

### Correlates of Latent Class Membership

Increasing age was significantly associated with membership in the “Polydrug-Socioeconomic Syndemic” class relative to the “Syndemic-free” class (OR = 1.29, 95% CI = 1.05, 1.60). Compared to those who were employed and/or enrolled in school, unemployed non-students were significantly more likely to fall in the “Polydrug-Socioeconomic Syndemic” class relative to the “Syndemic-free” class (OR = 5.10, 95% CI = 1.63, 15.91). Compared to those aged 21–24 years and those without insurance, participants aged 15–20 years (OR = 0.18, 95% CI = 0.03, 1.09; *p* = 0.062) and with insurance (OR = 0.37, 95% CI = 0.12, 1.18; *p* = 0.094) were marginally less likely to fall into the “Polydrug-Socioeconomic Syndemic” class relative to the “Syndemic-free” class. Compared to those not in school, participants currently in school were marginally less likely to fall in the “Polydrug-Socioeconomic Syndemic” class (OR = 0.36, 95% CI = 0.11, 1.15; *p* = 0.085) and marginally more likely to fall in the “Distress-Socioeconomic Syndemic” class (OR = 2.47, 95% CI = 0.98, 6.24; *p* = 0.056) relative to the “Syndemic-free” class (Table 3).

**Table 3:** Characteristics associated with syndemic class membership among YLWH in the US, 2019–2022 (*N* = 206)

	Polydrug-Socioeconomic vs. Syndemic-free	Distress-Socioeconomic vs. Syndemic-free
	OR (95% CI)	OR (95% CI)
Continuous age	1.29 (1.05, 1.60)^*^	1.06 (0.87, 1.28)
Age group		
21–24 years	Ref.	Ref.
15–20 years	0.18 (0.03, 1.09)^~^	0.93 (0.41, 2.10)
Gender identity		
Cis man	Ref.	Ref.
Cis woman	0.29 (0.04, 1.98)	1.11 (0.44, 2.79)
Trans, other	1.55 (0.30, 8.17)	1.88 (0.48, 7.39)
Race/ethnicity		
Non-Hispanic Black	Ref.	Ref.
Non-Hispanic other	1.77 (0.46, 6.73)	0.66 (0.14, 3.09)
Hispanic	1.29 (0.35, 4.69)	2.00 (0.82, 4.86)
Sexual identity		
Gay	Ref.	Ref.
Bisexual, other nongay-identities	2.30 (0.75, 7.04)	1.95 (0.74, 5.17)
Straight	0.11 (0.01, 3.08)	0.68 (0.24, 1.88)
Education		
Not currently in school	Ref.	Ref.
Currently in school	0.36 (0.11, 1.15)^~^	2.47 (0.98, 6.24)^~^
Employment status		
Employed and/or in school	Ref.	Ref.
Unemployed and not in school	5.10 (1.63, 15.91)^†^	1.58 (0.53, 4.71)
Insurance status		
Uninsured	Ref.	Ref.
Insured	0.37 (0.12, 1.18)^~^	1.32 (0.39, 4.45)
Relationship status		
Single	Ref.	Ref.
Partnered	0.85 (0.26, 2.78)	1.49 (0.64, 3.46)

YLWH: youth living with human immunodeficiency virus; US: United States; OR: odds ratio; aOR: adjusted odds ratio; CI: confidence interval; ~*p* < 0.10, ^*^p < 0.05, ^†^*p* < 0.01

Compared to those not enrolled in school, those enrolled in school were significantly more likely to fall in the “Distress-Socioeconomic Syndemic” class relative to the “Polydrug-Socioeconomic Syndemic” class (OR = 6.81, 95% CI = 1.63, 28.34). Compared to those aged 21–24 years, those aged 15–20 years were marginally more likely to fall in the “Distress-Socioeconomic Syndemic” class relative to the “Polydrug-Socioeconomic Syndemic” class (OR = 5.21, 95% CI = 0.73, 36.95; *p* = 0.099; not displayed).

### HIV Care Outcomes by Latent Class

**Fig. 2:**
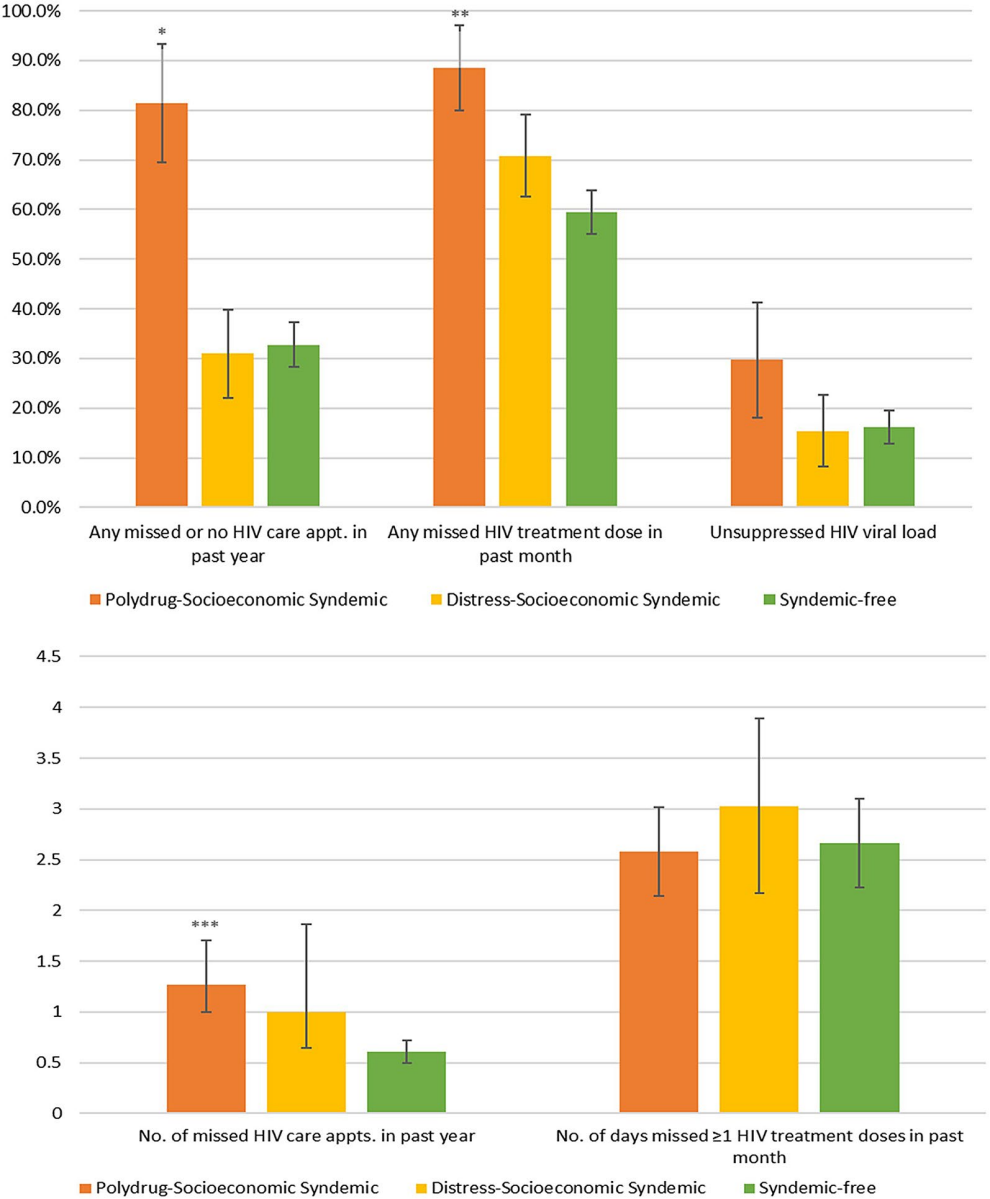
HIV care outcomes by latent class among YLWH in the US, 2019–2022 (*N* = 206) YLWH: youth living with human immunodeficiency virus; US: United States *Significantly higher than “Syndemic-free” (*p* < 0.001) and “Distress-Socioeconomic” (*p* < 0.01) classes; **significantly higher than “Syndemic-free” class (*p* < 0.01); ***significantly higher than “Syndemic-free” class (*p* < 0.05)

Having missed or had no HIV care appointments in the past year was most prevalent in the “Polydrug-Socioeconomic Syndemic” class (81.4%), which was significantly higher than that found in the “Distress-Socioeconomic Syndemic” (31.0%; χ^2^=10.49, df = 1, *p* = 0.001) and “Syndemic-free” classes (32.8%; χ^2^=13.62, df = 1, *p* < 0.001). Mean number of missed HIV care appointments in the past year was likewise highest in the “Polydrug-Socioeconomic Syndemic” class (1.27 missed appointments), which was significantly higher than that found in the “Syndemic-free” class (0.61 missed appointments; χ^2^=4.71, df = 1, *p* = 0.030) but not the “Distress-Socioeconomic Syndemic” class (1.00 missed appointments; χ^2^=0.34, df = 1, *p* = 0.562). Having missed any HIV treatment dose in the past month was most prevalent in the “Polydrug-Socioeconomic Syndemic” class (88.5%), which was significantly higher than that found in the “Syndemic-free” class (59.4%; χ^2^=8.32, df = 1, *p* = 0.004) but not the “Distress-Socioeconomic Syndemic” class (70.8%; χ^2^=2.00, df = 1, *p* = 0.158). There were no between-class differences with regard to mean number of days ≥ 1 HIV treatment doses were missed in the past month or with regard to unsuppressed HIV viral load prevalence. However, regarding the latter, prevalence was nearly twice as high in the “Polydrug-Socioeconomic Syndemic” class (29.7%) relative to the “Syndemic-free” (16.2%) and “Distress-Socioeconomic Syndemic” classes (15.4%; Fig. 2).

## Discussion

We determined the extent to which psychosocial factors co-occurred to form latent syndemic classes among racially/ethnically diverse YLWH, identified correlates of latent class membership, and documented variation in HIV care continuum outcomes across classes. We found two syndemic classes (“Polydrug-Socioeconomic Syndemic,” “Distress-Socioeconomic Syndemic”), which featured numerous co-occurring psychosocial conditions, and one “Syndemic-free” class, which featured no co-occurrence. Prior research with adults living with HIV has documented two to five syndemic classes [38–40, 61–63], typically including one large syndemic-free class and multiple smaller syndemic classes [39, 40, 62, 63], reflecting our findings. Age, education, employment, and insurance status were associated with class membership to varying degrees, adding evidence to the existence of these classes as valid patterns of co-occurring psychosocial factors in this sample. HIV care engagement and treatment adherence significantly differed between classes, and unsuppressed viral load non-significantly differed.

Smallest in prevalence, the “Polydrug-Socioeconomic Syndemic” class depicted perhaps the most severe syndemic, featuring 6/9 co-occurring conditions: marijuana misuse, alcohol misuse, illicit drug misuse, legal involvement, and food and housing insecurity. Moreover, endorsement probabilities for all 6 conditions were the highest in this class compared to the other classes. Similar syndemic patterns have emerged among women with HIV in Canada and among Ryan White HIV/AIDS Program clients in Miami, Florida [61, 63]. Alcohol and marijuana misuse most strongly distinguished the “Polydrug-Socioeconomic Syndemic” from the “Distress-Socioeconomic Syndemic” class. Polydrug use has been commonly found in adolescent populations, often older adolescents [64], and we did find that older age was associated with “Polydrug-Socioeconomic Syndemic” class membership. That the “Polydrug-Socioeconomic Syndemic” class fared worse on all HIV care continuum outcomes is not surprising given its syndemic and reflects prior studies with adults in which classes featuring drug misuse and socioeconomic insecurity were most strongly linked to adverse HIV or other outcomes [38, 40, 61, 63].

The “Distress-Socioeconomic Syndemic” class also showed a severe syndemic, featuring 6/9 co-occurring conditions (clinical depression and anxiety, enacted HIV stigma, marijuana misuse, food insecurity, legal involvement). Of the 3 classes, endorsement probabilities for depression, anxiety, and stigma were highest in this class. Classes featuring mental distress have been previously identified among African-American adults living with HIV in Baltimore, Maryland [40], and classes featuring HIV stigma have been previously identified among women living with HIV in Canada [61]. Clinical depression and anxiety most strongly distinguished the “Distress-Socioeconomic Syndemic” class from the “Polydrug-Socioeconomic Syndemic” class. Both forms of mental distress disproportionately occur among YLWH [65] and were likely elevated during data collection (during the COVID-19 pandemic) due to youth’s being unable to attend school with peers [66]. Indeed, participants enrolled in school tended to fall into this class. Of course, mental distress could have been elevated for those not in school as well, secondary to isolation, socioeconomic difficulties, and other issues due to COVID-19 conditions [66].

This mental health burden is concerning given inadequate access to mental health resources for YLWH, especially for racial/ethnicity minority YLWH [65]. Mental distress will likely complicate – and be further exacerbated by – the transition to adult HIV care [67, 68]. HIV treatment nonadherence and missed HIV care appointments were non-significantly higher in the “Distress-Socioeconomic Syndemic” relative to the “Syndemic-free” class. Prior research has documented statistically significant negative associations between classes featuring mental distress and HIV viral suppression [40, 63], though samples used in these prior studies were larger than ours and comprised of adults rather than youth.

Socioeconomic vulnerabilities, particularly economic insecurity and legal involvement, were common in both syndemic classes. This is a reminder of the extent to which racial/ethnicity minorities (including youth) are disproportionately impacted by poverty and the justice system in the US [69, 70]. Such factors may present competing priorities, especially in conjunction with mental distress or substance use, that take precedence over and further complicate HIV care engagement, retention, and treatment adherence. Uninsured and unemployed nonstudent participants tended to fall into the “Polydrug-Socioeconomic Syndemic” class, reflecting how socioeconomic vulnerabilities were elevated for this group.

Notably, HIV outcomes were suboptimal for the “Syndemic-free” class as well. Though there was no evidence of a syndemic in this class, marijuana misuse was elevated. Whether this could account for suboptimal HIV care continuum outcomes in this class is unclear, though there is some evidence that marijuana use, especially recreational (vs. therapeutic), can affect adherence to HIV treatment [7, 71, 72]. Unmeasured syndemic factors, such as violence, childhood abuse, and mental health sequelae (e.g., posttraumatic stress), as well as structural, community, and interpersonal forms of racism, could have also played a role.

### Limitations

Our findings should be considered in light of several limitations. Our cross-sectional data prevent the establishment of causality between our syndemic classes and HIV outcomes. Our sample was relatively small, urban, geographically diverse, and comprised of various racial, ethnic, sexual, and gender identities. Moreover, our sample was restricted to participants with access to technology (i.e., continuous internet access, email access, and SMS messaging for the intervention period), precluding participation by YLWH without such access and who may indeed be among the most marginalized and underserved. These issues may have affected the generalizability of our findings. We did not collect data on other potentially consequential psychosocial factors (e.g., racial/ethnic stigmas, violence) that may co-occur with the conditions we examined, which may have resulted in an incomplete picture of the challenges facing YLWH in our sample. Examination of other factors in addition to the ones we examined may reveal a number and composition of classes, and therefore distribution of HIV outcomes across classes, that differ from our findings. Finally, the majority of data collection occurred in the midst of the COVID-19 pandemic, during which YLWH could have experienced elevated substance use, anxiety, and depression. Therefore, results should be replicated during non-pandemic times.

### Research and Practice Implications

Given the dearth of research examining syndemic psychosocial patterns among YLWH, more studies with this population using similar analytic approaches are warranted. Such scholarship can help establish how common certain syndemic patterns are among YLWH. Additional research can also increase understanding on the interactive and potentially causal nature of commonly experienced psychosocial conditions among YLWH, such as the use of drugs or the experience of mental distress as responses to socioeconomic insecurities. Further research is needed to identify whether syndemic psychosocial conditions work in concert to shape HIV outcomes and require intervention approaches that target all conditions, or whether there are priority conditions that exert greater influence in shaping HIV outcomes and therefore merit narrower, more targeted interventions. Also, research to examine whether syndemic patterns are proxies for broader community, environmental, and structural disadvantage that shapes psychosocial syndemics would be helpful.

Addressing psychosocial syndemics necessitates tailored intervention and programming across multiple avenues. Improved, developmentally-tailored screening and treatment for substance misuse and mental distress are warranted, ideally integrated into extant systems of HIV care. However, community-/family-based interventions [73–75] may be important to implement or retain as alternatives due to medical mistrust and healthcare-related racism and discrimination. Novel approaches to treat substance use and mental distress could be incorporated as part of broader youth-/patient-centered HIV care models [76–78]. Youth-friendly technology-based treatment delivery modalities (e.g., video-conferencing/counseling, cellphone support), which have shown promise [79–81], could help address access and stigma-related barriers. Syndemic-focused, transdiagnostic treatment approaches may also be considered [82].

Connecting socioeconomically vulnerable youth to organizations that participate in programs like the Housing Opportunities for Persons with AIDS Program [83] and Workforce Innovation and Opportunity Act’s Youth Activities Formula Grant Program or YouthBuild [84] can help youth access housing, education, vocational training, mentorship, and employment that prior legal involvement may otherwise hamper. Indeed, economic interventions can effectively support HIV care continuum progress [85]. In sum, increased focus on the social determinants of health to better serve YLWH is needed. Comprehensive, holistic interventions may show the greatest promise for improving the cascade of care for YLWH in the US.

## Data Availability

De-identified data from this study are not available in a public archive but may be requested by emailing KJH.

## Code Availability

Analytic code used to conduct the analyses in this study are not available in a public archive but may be requested by emailing JMW.
